# Expression of Functional Molecule on Plasmacytoid Dendritic Cells Is Associated With HBsAg Loss in HBeAg-Positive Patients During PEG-IFN α-2a Treatment

**DOI:** 10.3389/fimmu.2022.891424

**Published:** 2022-05-19

**Authors:** Weihua Cao, Si Xie, Lu Zhang, Xiaoyue Bi, Yanjie Lin, Liu Yang, Yao Lu, Ruyu Liu, Min Chang, Shuling Wu, Ge Shen, Jianping Dong, Yao Xie, Minghui Li

**Affiliations:** ^1^ Department of Hepatology Division 2, Beijing Ditan Hospital, Capital Medical University, Beijing, China; ^2^ Department of Infectious Diseases, Miyun Teaching Hospital, Capital Medical University, Beijing, China; ^3^ Division of Hepatology, Hepato-Pancreato-Biliary Center, Beijing Tsinghua Changgung Hospital, School of Clinical Medicine, Tsinghua University, Beijing, China; ^4^ Department of Hepatology Division 2, Peking University Ditan Teaching Hospital, Beijing, China; ^5^ Department of Infectious Diseases, Haidian Hospital, Beijing Haidian Section of Peking University Third Hospital, Beijing, China

**Keywords:** hepatitis B surface antigen, chronic hepatitis B, interferon, functional cure, plasmacytoid dendritic cells

## Abstract

**Objective:**

The ideal endpoint of antiviral therapy in chronic hepatitis B (CHB) patients is to clear hepatitis B surface antigen (HBsAg). This study aimed to evaluate whether the expression of functional molecules on plasmacytoid dendritic cells (pDCs) is associated with HBsAg loss in HBeAg-positive patients during peginterferon alpha-2a (PEG IFN α-2a) therapy.

**Methods:**

A single-center prospective cohort study was performed in HBeAg-positive CHB patients who were treated with PEG-IFN α-2a and followed up for 4 years. HBsAg clearance, HBeAg loss and undetectable HBV DNA achieved by PEG-IFN α-2a therapy was considered as functional cure. The frequencies of pDC and CD86^+^ pDC in peripheral blood, and the mean fluorescence intensity of CD86 (CD86MFI) on the surface of pDC were measured at starting therapy, after 12 and 24 weeks of therapy.

**Results:**

Of 63 patients enrolled, 17 patients achieved HBsAg loss. The baseline HBV DNA load in Non-functional-cure group was significantly higher than that in Functional cure group, and the CD86^+^ pDC% was significantly lower in patients without functional cure. HBV DNA load (OR=0.146, *P* = 0.002) and CD86^+^ pDC% (OR=1.183, *P* = 0.025) were independent factors associated with functional cure confirmed by binary logistic regression analysis. In the Functional cure group, HBsAg, HBeAg, and HBV DNA loads decreased remarkably after 12 weeks and 24 weeks of treatment compared to baseline. In Non-functional-cure group, CD86^+^ pDC% and CD86MFI increased significantly from baseline after 12 weeks of treatment. In the Functional cure group, compared with baseline, pDC% increased significantly at 24 weeks, while CD86MFI increased significantly after 24 weeks of treatment.

**Conclusion:**

The lower the baseline HBV DNA load and the more the baseline CD86^+^ pDC%, the easier it is for patients to obtain functional cure.

## Introduction

About 250-300 million people are chronically infected with HBV (1, 2). Chronic hepatitis B (CHB) remains an important worldwide public health problem, and long-term chronic infection can result in liver cirrhosis, hepatic failure and hepatocellular carcinoma (3). There is an urgent need to explore optimized therapeutic strategy for CHB. Moreover, antiviral therapy is the most effective treatment for slowing down the progression of CHB. Currently, “functional cure” defined as a sustained HBsAg loss and undetectable HBV DNA, with or without HBsAg seroconversion in serum, is the endpoint of antiviral treatment for CHB (4, 5). In the natural process of chronic HBV infection, HBsAg loss is as rare as 1.15% per year (6). Several studies indicated that degradation or permanent inactivation of cccDNA can contribute to achieving the “functional cure” of CHB (7–9). In the current antiviral therapies, peginterferon alpha-2a (PEG IFN α-2a), as a first-line antiviral drug, has direct antiviral as well as immunomodulatory activity (10, 11). PEG-IFN-a therapy for CHB can result in HBeAg clearance, HBsAg loss and even HBsAg seroconversion in about 10%-30% patients within a certain period of time (12–15).

In addition to the direct antiviral activity, the immunomodulatory effect of IFN-α may eventually induce an immunological control of hepatitis B infection (10, 11). As a vital component of early antiviral innate immune response, plasmacytoid dendritic cells (pDCs) are the main effector cells producing IFN α *in vivo* in response to viral infection (16). In chronic HBV infection, pDCs’ function is markedly impaired by hepatitis B virology (17–19). HBsAg blocks the IRF7 phosphorylation signal pathway induced by TLR9, resulting in decrease of IFN-α generation and impaired expression of costimulatory molecules (such as CD80, CD86, CD83) on the surface of pDCs (17, 20, 21). Martinet et al. have proven that pDCs with defective responses to stimulation with Toll-like receptor 9 ligand (TLR9-L) did not induce cytolytic activity of NK cells in hepatitis B patients (22). Other study revealed that, acting as an immune tolerant protein, HBeAg may promote DCs differentiation into regulatory dendritic cells (DCregs) which can induce naive T cells to differentiate into Tregs to impair virus-specific cytotoxic T lymphocyte (CTL) response (23). Therefore, pDCs dysfunction may be a crucial cause for the persistence of hepatitis B infection. Active immunotherapy based on pDCs may become a prospective method to treat CHB.

Previous studies suggested that PEG-IFN-α can activate pDCs in the early stage of chronic hepatitis B (24–26). These studies illustrated the changes of immune cell function in the process of interferon treatment of CHB, but little is known about the frequency and functional changes of pDCs in CHB patients with functional cure after PEG-IFN α treatment. Therefore, this study aims to explore whether the frequency of pDCs and the expression of functional molecules on pDCs are associated with HBsAg loss in HBeAg-positive CHB patients during PEG-IFN α-2a therapy.

## Materials and Methods

### Patients

This prospective study was performed in the Liver Disease Center of Beijing Ditan Hospital, Capital Medical University between October 2014 and October 2017, and 63 HBeAg-positive CHB patients as naïve therapy were enrolled. The ethical approval of this study was obtained from the Ethics Review Committee of Beijing Ditan Hospital of Capital Medical University. All participants provided written patients’ informed consent for sample collection and subsequent follow-up. This study was registered at clinicaltrials.gov (Clinical Trials. gov ID: NCT03210506).

The enrollment criteria were: patients with HBsAg and HBeAg positivity for more than 6 months, detectable HBV DNA, and abnormal alanine aminotransferase (ALT) levels lasting for at least 3 months, or liver biopsy revealing significant inflammation. The exclusion criteria were: 1) patients co-infected with other viruses such as HIV, HDV, HCV; 2) carrying HIV or syphilis antibodies; 3) accompanied with other liver diseases such as alcoholic liver disease, fatty liver, metabolic liver disease, autoimmune hepatitis, liver cirrhosis, or hepatic cancer; 2) using hormone or immunomodulatory medication during the 4-year follow-up period.

These patients were assigned to receive personalized PEG-IFN α-2a 180 μg subcutaneous injection weekly and complete a 4-year follow-up. These patients received PEG-IFN α‐2a monotherapy for 24 weeks. If HBV DNA turned negative, patients continued to receive PEG-IFN α‐2a monotherapy. Otherwise, patients received PEG-IFN α‐2a combined with entecavir or tenofovir therapy according to the patients’ past medication history or physical condition. In the subsequent treatment, if HBsAg and HBeAg declined continuously and even reached functional cure, patients continued to receive PEG-IFN α‐2a consolidation treatment for 12-24 weeks; however, if HBsAg and HBeAg did not continue to decline and remain in the plateau phase, PEG-IFN α‐2a was stopped and ETV or TDF single-agent maintenance therapy was continued. Serum HBV DNA loads, HBsAg/anti-HBs, HBeAg, and HBe antibody (HBeAb) levels were detected before treatment and thereafter every 12 weeks until HBsAg loss. The frequency and function of pDCs were measured before treatment as the baseline and at weeks 12 and 24 of treatment.

### Definition of Functional Cure

The American Association for the Study of Liver Diseases (AASLD) and the European Association for the Study of the Liver (EASL) set the hepatitis B therapy endpoints as functional cure, which is defined as sustained HBsAg loss assayed with a lower limit of HBsAg detection of 0.05 IU/mL, with or without HBsAg seroconversion, undetectable HBV DNA and the persistent transcriptional inactivation of cccDNA after the end of the treatment.

### Clinical Indicators Detection

The Roche Cobas AmpliPrep/Cobas TaqMan 96 full-automatic real-time fluorescent quantitative PCR detection reagent (Roche, Pleasanton, CA) was applied for the detection of serum HBV DNA loads; the Abbott Architect i2000 Detection Reagent (Abbott Diagnostics, Abbott Park, IL) was used to test Serum HBsAg/anti-HBs, HBeAg, and HBeAb levels; and Hitachi 7600 full-automatic biochemical analyzer was applied to measure the parameters of liver function.

### Analysis of pDCs by Flow Cytometry

**Figure 1:**
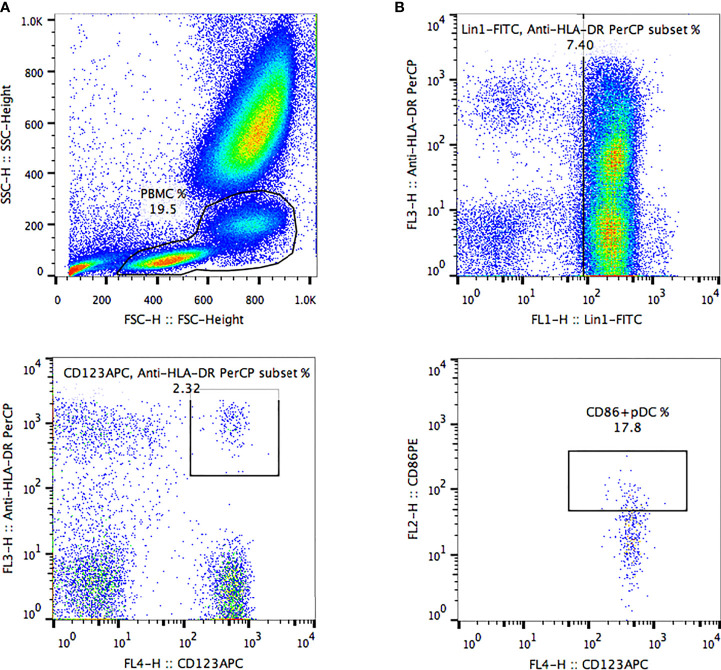
The frequency of peripheral blood pDCs and expression of costimulatory molecule CD86 were analyzed by FlowJo software. **(A)** Peripheral blood mononuclear cells were delineated according to forward scatter and side scatter. **(B, C)** pDCs were delineated based on anti-human HLA-DR, anti-human lineage cocktail, and anti-human CD123 in mononuclear cells. **(D)** Delineation of CD86^+^ pDCs was based on the CD86-PE marker in pDCs. HLA-DR+ CD123+ lineage-cells were defined as pDCs. The pDC proportion among peripheral blood mononuclear cells was defined as pDC frequency (pDC%). pDCs expressing costimulatory molecule CD86 on the surface were defined as CD86^+^ pDCs. The CD86^+^ pDC proportion among pDCs was defined as CD86^+^ pDC frequency (CD86^+^ pDC%). Quantification of pDCs included the frequencies of pDCs and CD86^+^ pDCs and the mean fluorescence intensity of CD86 (CD86MFI).

Peripheral venous blood samples from these patients were collected into EDTA anticoagulant tubes to analyze pDCs within 4 h. Three monoclonal antibodies (mouse anti-human leukocyte antigen (HLA)-DR (Clone L243), mouse anti-human CD123 (Clone 7G3) and mouse anti-human lineage cocktail 1 (Lin 1) (CD3, Clone SK7; CD14, Clone MøP9; CD16, Clone 3G8; CD19, Clone SJ25C1; CD20, Clone L27; CD56, Clone NCAM16.2) from BD Biosciences (San Jose, CA) were added into blood to label pDCs, then mouse anti-human CD86 (Clone 2331 (FUN1) from BD Biosciences (San Jose, CA) was added to label the costimulatory molecules CD86 on the surface of pDCs in the dark at room temperature for 20 min. Then 2 mL red blood cell lysis buffer (BD Biosciences) was evenly mixed into the above samples and left aside for 5 min in the dark at room temperature. Cell debris in the supernatant was removed by centrifugation at 1200 rpm for 5 min. Then, the supernatant was discarded and cells were washed with 2 mL of 1 x PBS which were spun (1200 rpm, 5 min). Finally, after discarding the supernatant and adding the 200 μL of 1 x PBS, pDCs were detected. pDCs were quantified by 4-color FACSCalibur (BD Biosciences) and FlowJo 7.6.1 software, as shown in **Figure 1**. The proportion of pDC in peripheral blood mononuclear cells was regarded as the frequency of pDC (pDC%). pDCs which express costimulatory molecule CD86 on the surface were regarded as CD86^+^ pDCs. The proportion of CD86^+^ pDC in pDCs was regarded as the frequency of CD86^+^ pDC (CD86^+^ pDC%). CD86^+^ pDC% and CD86MFI were used to reflect the function of pDC.

### Statistical Analysis

Data were performed using SPSS for Windows software, version 26 (SPSS Inc., Chicago, IL, USA), and Prism software (GraphPad Software version 5.01). All data were described as mean ± standard deviation (SD) or median (Q1, Q3). Changes of the parameters at different time points were analyzed by repeated-measures analysis of variance; Bonferroni was used to adjust *P* values for multiplicity, and the correction level was α. There were three observation time points for pDC/PBMC%, CD86^+^ pDC% and CD86MFI. α was set as 0.016, and *P*<0.016 was considered to be statistically significant. Comparisons between two groups were carried out Using the Mann-Whitney nonparametric *U* test. The association of variables was assessed by Spearman’s correlation. Independent factors of functional cure were analyzed by binary logistic regression analysis. All analyses of significance were two-tailed, and *P* value of less than 0.05 was considered as statistically significant.

## Results

### Characteristics of Enrolled Patients

In this study, 63 HBeAg positive CHB patients received PEG-IFN-α-2a personalized treatment and completed the 4-year follow-up. There were 38 males and 25 females, with an average age of 30 years (20-51 years). After PEG-IFN α-2a treatment, 17 cases achieved functional cure. Two patients in the Functional cure group and 6 patients in the Non-Functional-cure group were lost to follow-up at 12 or 24 weeks of treatment.

There was no significant difference in serum HBsAg, HBeAg, or ALT levels between the two groups at baseline, but HBV DNA load in Non-functional-cure group was significantly higher than that in Functional cure group (*P* = 0.007, **Table 1**). At the 12^th^ week and the 24^th^ week, HBsAg levels (12w *P*=0.000, 24w *P*=0.000), HBeAg levels (12w *P*=0.036, 24w *P*=0.016) and HBV DNA loads (12w *P*=0.014, 24w *P*=0.001) in Functional cure group were significantly lower than those in Non-functional-cure group as shown in **Table 1**. We analyzed the relationship between the dynamic changes of clinical indicators and HBsAg clearance by binary logistic regression, but found no correlation. For the frequency of pDC and the expression of costimulatory molecule CD86, the pDC% (*P* = 0.620) and CD86MFI (*P* = 0.114) were not different between the two groups, while Functional cure group had higher CD86^+^ pDC% (*P* = 0.037) than Non-functional-cure group (**Table 2**).

**Table 1:** Characteristics of subjects.

Item	All patients	Baseline		*P* ^*^ value	12w		*P* ^*^ value	24w		*P* ^*^ value
	N=63	Non-functional-cure group	Functional cure group		Non-functional-cure group	Functional cure group		Non-functional-cure group	Functional cure group	
		N = 46	N=17		N = 40	N=15		N = 40	N = 15	
Male/Female	38/25	30/16	8/9		25/15	6/9		25/15	6/9	
Age (yrs)	30 (20–51)	30 (20–51)	32 (24–49)	0.625	30 (20–51)	32 (24–49)	0.857	30 (20–51)	32 (24–49)	0.857
HBsAg level (log10 IU/mL)	3.857 ± 0.692	3.905 ± 0.687	3.727 ± 0.710	0.345	3.377 ± 0.702	1.797 ± 1.460	0.000	3.196 ± 0.802	1.502 ± 1.654	0.000
HBV DNA load (log_10_ IU/mL)	7.059 ± 1.161	7.330 ± 0.935	6.324 ± 1.405	0.007	4.095± 1.555	2.922 ± 1.447	0.014	2.084± 0.760	1.233 ± 0.879	0.001
HBeAg concentration (PEIU/mL)	902.260 (411.970, 1403.620)	916.210 (371.640, 1396.338)	880.570 (441.715, 1409.685)	0.975	59.895 (16.438, 406.53)	29.94 (0.36, 124.35)	0.036	18.92 (5.728, 82.75)	1.43 (0.33, 32.36)	0.016
ALT level (U/L)	253.300 (129.600, 361.100)	240.700 (128.350, 361.300)	273.000 (135.250, 371.150)	0.361	67.15 (49.45, 82.05)	48.30 (20.70, 86.80)	0.186	44.25 (28.15, 56.425)	26.90 (22.40, 64.50)	0.177

^*^Non-functional-cure group vs. Functional cure group.

**Table 2:** Baseline pDC frequency and expression of costimulatory molecule CD86 between two groups.

Item	All patients (n = 63)	Non-functional-cure group (n = 46)	Functional cure group (n = 17)	*P* value^*^
Male/Female	38/25	30/16	8/9	
pDC/PBMC	23.601 ± 11.116	24.414 ± 12.001	21.767 ± 8.276	0.620
CD86^+^ pDC (%)	22.543 ± 8.203	22.109 ± 10.599	25.948 ± 6.542	0.037
CD86MFI	61.000 (49.600, 79.900)	62.600 (49.900, 80.300)	56.500 (47.000, 65.400)	0.114

^*^Non-functional-cure group vs. Functional cure group.

Binary logistic regression was used to analyze the correlation of baseline serological and virological indexes, pDC frequency and CD86 expression with the occurrence of HBsAg loss after PEG-IFN therapy. Results showed that HBV DNA load (OR = 0.146, 95% CI (0.044, 0.489), *P* = 0.002) and CD86^+^ pDC% (OR = 1.183, 95% CI (1.021, 1.370), *P* = 0.025) were two independent factors associated with functional cure.

### Correlations Between pDCs and HBV Virological Characteristics, ALT Before PEG-IFN Therapy

**Figure 2:**
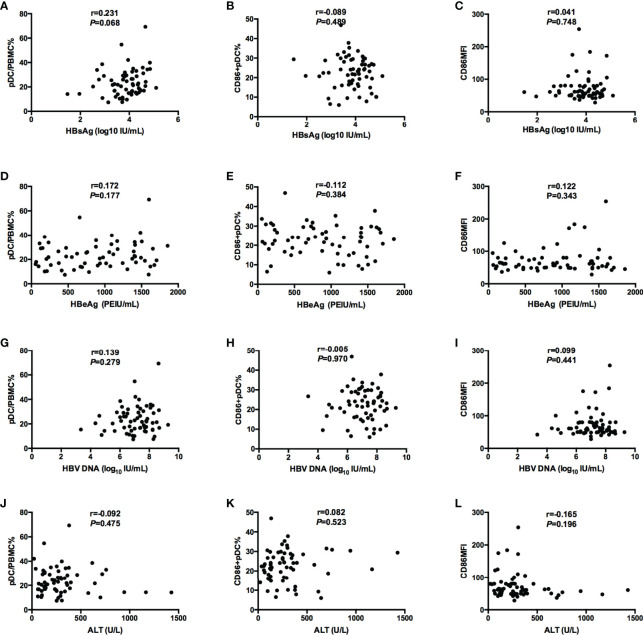
Correlations between pDC frequency or function (pDC%, CD86+ pDC%, and CD86MFI) and serological and virological indicators (HBsAg, HBeAg, and HBV DNA, ALT). **(A–C)** Correlations between pDC frequency or function [pD% **(A)**, CD86+ pDC% **(B)**, and CD86MFI **(C)**] and HBsAg. **(D–F)** Correlations between pDC frequency or function [pDC% **(D)**, CD86+ pDC% **(E)**, and CD86MFI **(F)**] and HBeAg. **(G–I)** Correlations between pDC frequency or function [pDC% **(G)**, CD86+ pDC% **(H)**, and CD86MFI **(I)**] and HBV DNA. **(J–L)** Correlations between pDC frequency or function [pDC% **(J)**, CD86+ pDC% **(K)**, and CD86MFI **(L)**] and ALT.

Before treatment, there was no correlations between the frequency of pDC and the expression of surface costimulatory molecule CD86 with the baseline virological markers, including HBV DNA load, HBsAg level and HBeAg concentration (**Figure 2**).

### PDC Function and Treatment Response During PEG-IFN- α Treatment

**Figure 3:**
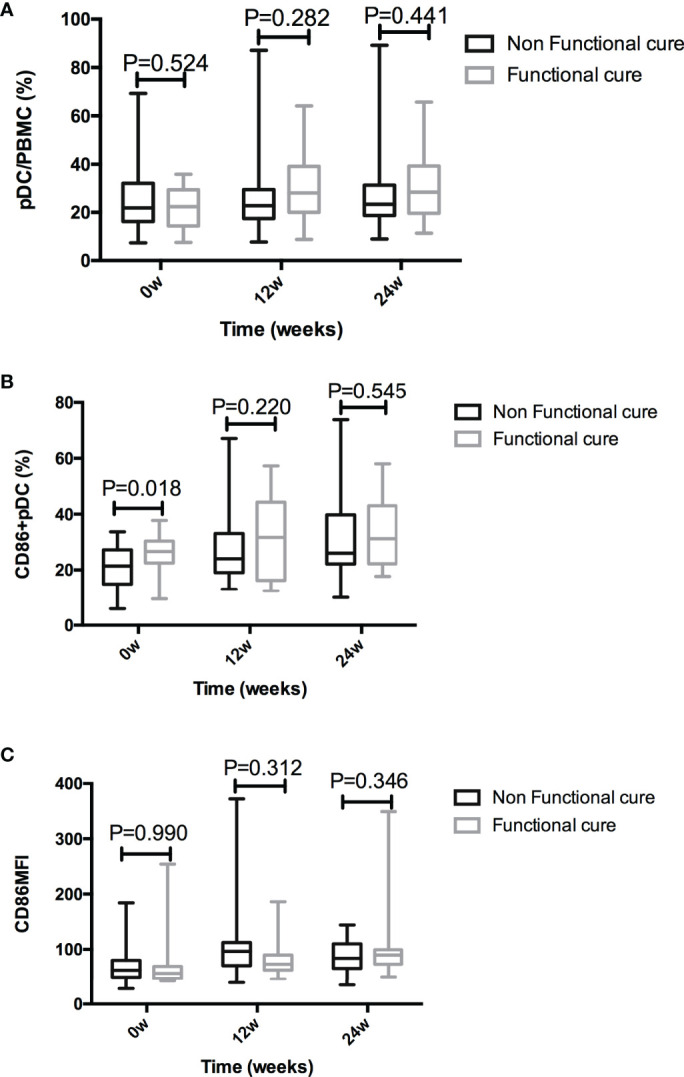
Comparison of pDC% **(A)**, CD86+ pDC% **(B)**, and CD86MFI **(C)** between the Functional cure group and Non-functional-cure group at baseline and after PEG-IFN-a-2a therapy for 12 and 24 weeks.

The frequency of pDC were not significantly different between two groups at baseline (*P* = 0.524), 12 weeks (*P* = 0.282), or 24 weeks (*P* = 0.441) after treatment. The baseline CD86^+^ pDC% in Non-functional-cure group was significantly lower than that in Functional cure group (*P* = 0.018); however, there were no difference at the 12^th^ week and the 24^th^ week between two groups. The CD86MFI on the pDC surface were similar between two groups throughout the treatment (**Figure 3**). The baseline *P* values in **Table 2** and **Figure 3** were different due to the number of patients. **Table 2** showed the data of total number of patients. **Figure 3** showed the statistical analysis after excluding the lost follow-up patients (2 in the Functional cure group, 6 in the Non-functional-cure group).

**Figure 4:**
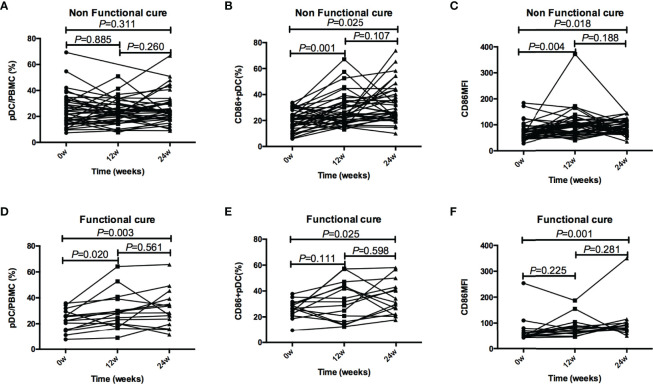
Tendencies of pDC frequency and function, including pDC%, CD86+ pDC%, and CD86MFI, at baseline and after 12 and 24 weeks of treatment in the Functional cure group and Non-functional-cure group. **(A–C)** Tendencies of pDC% **(A)**, CD86+ pDC% **(B)**, and CD86MFI **(C)** at baseline and after 12 and 24 weeks of treatment in the Non-functional-cure group. **(D–F)** Tendencies of pDC% **(D)**, CD86+ pDC% **(E)**, and CD86MFI **(F)** at baseline and after 12 and 24 weeks of treatment in the Functional cure group. The correction level a was set as 0.016, P < 0.016 was considered to be statistically significant.

In addition, compared with baseline, the frequency of pDC in Non-functional-cure group did not change after PEG-IFN α-2a treatment, while CD86^+^ pDC% and CD86MFI increased significantly after 12 and 24 weeks of treatment. In Functional cure group, the frequency of pDC and CD86^+^ pDC, and CD86MFI increased significantly from baseline after treatment (**Figure 4**).

We analyzed the correlations between pDC frequency or function and serological and virological indexes respectively in Non-functional-cure group and Functional cure group in 12 weeks and 24 weeks, but found no correlations between them (**Figures S1**, **S2**). There were no correlations between the levels of CD86^+^ pDC% and HBV viral load at week 12 and 24 for the Functional cure and Non-functional-cure group either (**Figure S3**).

## Discussion

Although pDCs exist as a small proportion of the total cells in blood, they are the main effector cells producing type I interferon *in vivo* and play a vital role in HBV infection. However, functional defects in pDCs have been reported in patients with chronic HBV infection (18, 19, 27). At present, the ultimate goal of CHB treatment is to achieve functional cure, which requires antiviral immune response. PEG-IFN-α has not only antiviral effect, but also immunomodulatory function (10, 11). Based on the above theory, we dynamically described the changes of pDCs in patients with CHB in the early stage of PEG-IFN-α-2a treatment, and explored whether the frequencies of pDCs and expression of functional molecules on the surface of pDCs are correlated with functional cure of CHB patients with HBeAg positivity during PEG-IFN α-2a therapy. Our study highlighted the function of pDCs in achieving functional cure and brought a fresh perspective of immunologic changes triggered by PEG-IFN α therapy in HBeAg positive CHB patients.

Our data showed that baseline HBV DNA load in patients with functional cure was significantly lower than that in patients without functional cure. The results of binary logistic regression demonstrated that baseline HBV DNA load was an independent influencing factor of functional cure, which is in line with reports indicating that the lower the viral load, the easier to obtain functional cure (28, 29). The viral load is at a relatively low level, and its transcription and replication activity is low, resulting that the production of viral protein is reduced, which is consistent with our result: virological indicators in patients with functional cure significantly decreased during PEG-IFN α treatment. Nevertheless, previous studies revealed that low baseline HBsAg was more reliable than serum HBV DNA levels for predicting good response to antiviral treatment in HBeAg-positive patients (30–34). Actually, the decrease of HBsAg indicates the decrease of cccDNA (35, 36), which is important for predicting the outcome of PEG-IFN α treatment in CHB patients. Theoretically, low baseline HBsAg is more likely to achieve HBsAg loss, but our data suggested that there was no significant difference in baseline HBsAg levels between the two groups, which may be due to the small sample size of our study.

HBV itself impairs the function of pDCs. As a bridge linking innate immunity and adaptive immunity, pDCs play a critical role in the immune response to HBV infection, such as producing type I interferon, antigen-presenting, and stimulating NK cells, T cells and other immune responses mediated by adaptive T cells (37–39). The function defect of pDCs may lead to deficiency of HBV immune elimination and persistent chronic infection. Actually, an effective antiviral immune response requires the involvement of functional pDCs. These functions of pDCs depend on maturation and activation of pDCs. A variety of costimulatory molecules are highly expressed on the surface of pDCs. CD86, as one of the costimulatory molecules, promotes the maturation and activation of pDCs. We analyzed the expression of CD86 during the early antiviral therapy in HBeAg (+) CHB patients to indirectly evaluate pDCs function. Our data suggested that the baseline CD86^+^ pDC% in patients with functional cure was higher than that in patients without functional cure, and the baseline CD86^+^ pDC% was an independent influencing factor of functional cure. At baseline, there was a significant difference in CD86^+^ pDC% between Functional cure group and Non-functional-cure group, indicating a difference in immune basis between them. The differences between the two groups at 12 and 24 weeks of treatment became insignificant owning to PEG-IFN treatment. The more the baseline CD86^+^ pDC%, the easier it is for patients to obtain functional cure. Moreover, after PEG-IFN α treatment, CD86 expression increased significantly, especially in patients with functional cure. Based on these results, it may be concluded that the increase in CD86 expression manifested the enhancement of pDCs function, which in turn promotes HBV clearance and even HBsAg loss.

In summary, our data shed new light on the correlation between HBsAg loss and functional changes of pDCs in the early PEG-IFN α treatment of HBeAg positive CHB patients. We observed that the lower the HBV DNA load, the more the frequency of CD86^+^ pDC before PEG-IFN-α treatment. The increase in frequency of pDC and the higher expression of functional molecules CD86 after PEG-IFN α treatment are more helpful for HBsAg loss. Generally, HBsAg loss/functional cure depends on the low titer HBV DNA load, the increase in the number of pDCs, and the recovery of function in the early stage of PEG-IFN α therapy. The findings could help provide a new therapeutic strategy for chronic hepatitis B in the pursuit of functional cure.

## Data Availability Statement

The raw data supporting the conclusions of this article will be made available by the authors, without undue reservation.

## Ethics Statement

The studies involving human participants were reviewed and approved by Beijing Ditan Hospital Research Ethics Committee. The patients/participants provided their written informed consent to participate in this study.

## Author Contributions

WC, SX, ML, YX, and JD conceived and designed this research. LZ, XB, YLi, and LY conducted experiments and collected the data. YLu, LZ, RL, MC, SW, and GS collected the sample information of the patients and ordered reagents and materials. WC performed the statistical results and wrote the manuscript with assistance from YX, ML, and SX. The final manuscript was approved by all authors.

## Funding

This research was supported by Beijing Hospitals Authority Clinical Medicine Development of Special Funding Support (No. XMLX 201706 and XMLX 202127), Special Public Health Project for Health Development in Capital (2021-1G-4061 and 2022-1-2172), the Digestive Medical Coordinated Development Center of Beijing Hospitals Authority (No. XXZ0302 and XXT28), the National Science and Technology Major Project of China (No. 2017ZX10201201-001-006 and 2017ZX10201201-002-006, and 2018ZX10715-005-003-005), and Beijing Municipal Science & Technology Commission (No. Z151100004015122, No. D161100002716002).

## Conflict of Interest

The authors declare that the research was conducted in the absence of any commercial or financial relationships that could be construed as a potential conflict of interest.

The Reviewer LJ declared a shared parent affiliation with the authors WC, LZ, XB, YL, LY, YJL, RL, MC, SW, GS, YX, and ML to the handling editor at the time of review.

## Publisher’s Note

All claims expressed in this article are solely those of the authors and do not necessarily represent those of their affiliated organizations, or those of the publisher, the editors and the reviewers. Any product that may be evaluated in this article, or claim that may be made by its manufacturer, is not guaranteed or endorsed by the publisher.

## Supplementary Material

Supplementary Figure 1 The correlations between pDC frequency or function and serological and virological indexes in Functional cure group in 12 weeks and 24 weeks. **Δ**: the dynamic changes of virological and clinical indicators from baseline to 12 weeks or from baseline to 24 weeks.

Supplementary Figure 2 The correlations between pDC frequency or function and serological and virological indexes in Non-functional-cure group in 12 weeks and 24 weeks. **Δ**: the dynamic changes of virological and clinical indicators from baseline to 12 weeks or from baseline to 24 weeks.

Supplementary Figure 3 The association between the levels of CD86^+^ pDC% and HBV viral load at week 12 and 24 for the Functional cure and Non-Functional cure group.
