# Supplementary figures and images for: Expression of Functional Molecule on Plasmacytoid Dendritic Cells Is Associated With HBsAg Loss in HBeAg-Positive Patients During PEG-IFN α-2a Treatment
Source: Front Immunol. 2022 May 19;13:891424. doi: 10.3389/fimmu.2022.891424 (PMC9160736; doi:10.3389/fimmu.2022.891424)

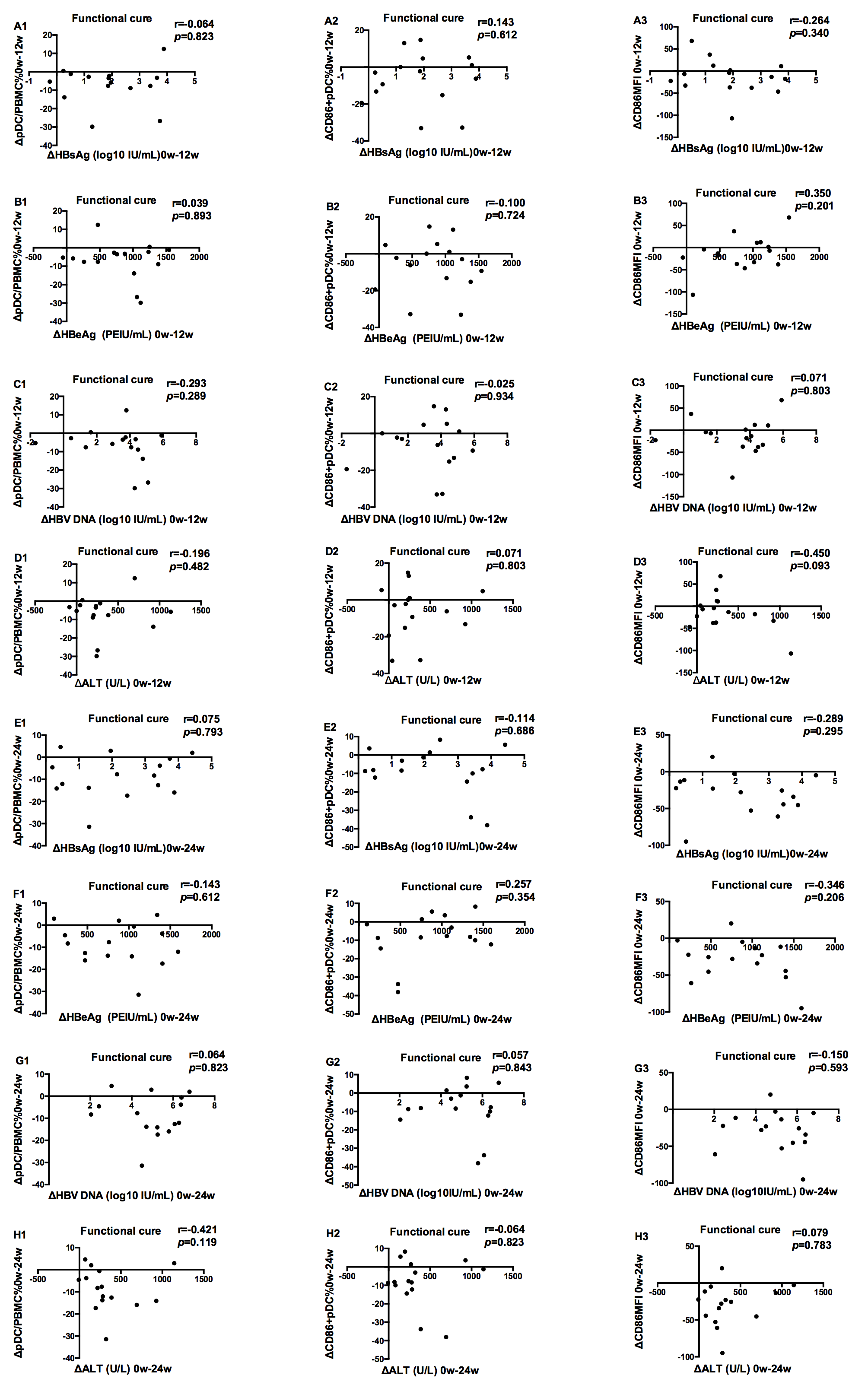

Supplement: Supplementary Figure 1 — The correlations between pDC frequency or function and serological and virological indexes in Functional cure group in 12 weeks and 24 weeks. Δ: the dynamic changes of virological and clinical indicators from baseline to 12 weeks or from baseline to 24 weeks. [file Image_1.tiff]

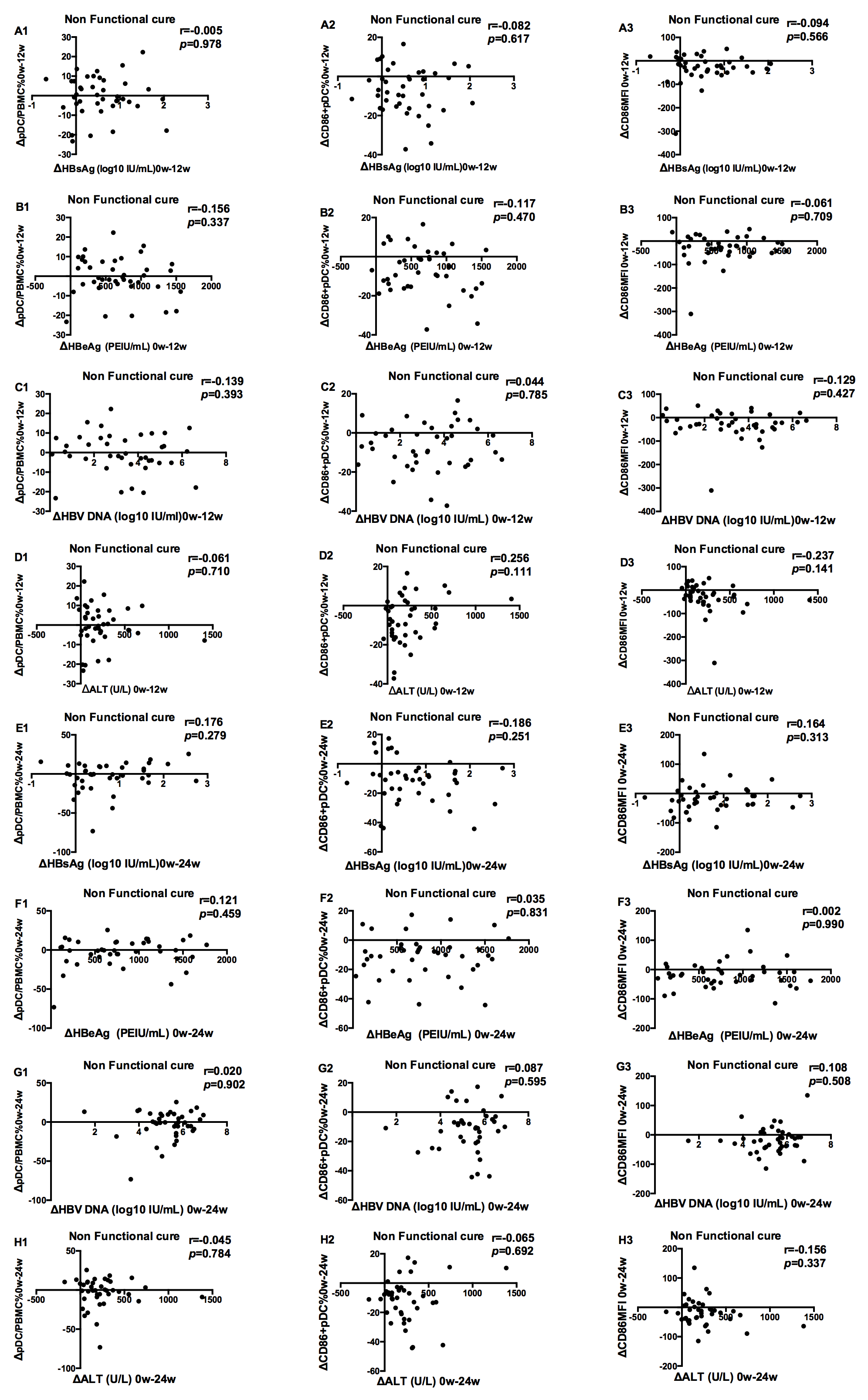

Supplement: Supplementary Figure 2 — The correlations between pDC frequency or function and serological and virological indexes in Non-functional-cure group in 12 weeks and 24 weeks. Δ: the dynamic changes of virological and clinical indicators from baseline to 12 weeks or from baseline to 24 weeks. [file Image_2.tiff]

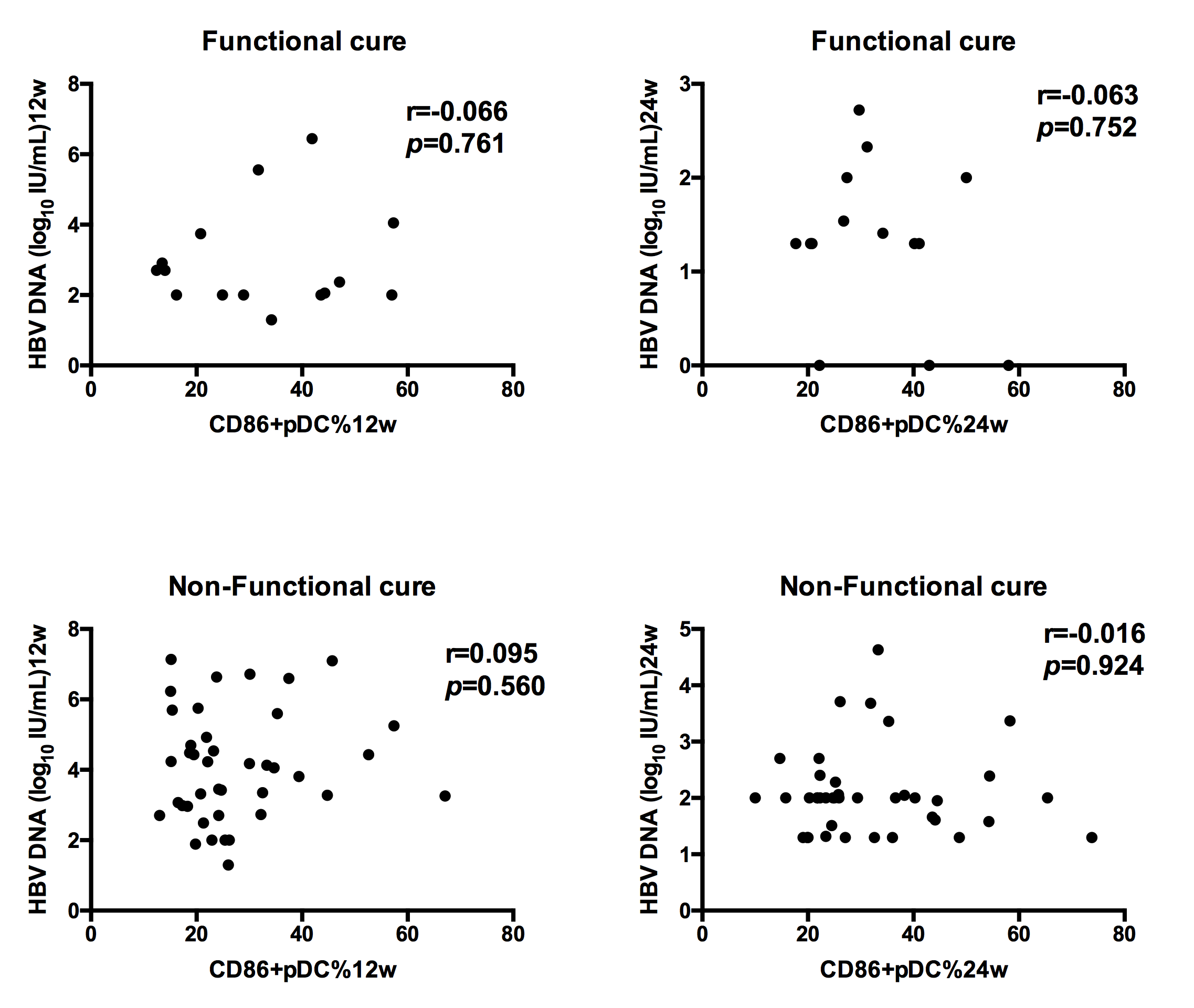

Supplement: Supplementary Figure 3 — The association between the levels of CD86+ pDC% and HBV viral load at week 12 and 24 for the Functional cure and Non-Functional cure group. [file Image_3.tiff]
